# Supplementary material for: Biochemical Composition and Alkaline Extraction Optimization of Soluble Bioactive Compounds from the Green Algae Caulerpa cylindraceae
Source: Mar Drugs. 2025 May 14;23(5):208. doi: 10.3390/md23050208 (PMC12113460; doi:10.3390/md23050208)
Supplement: Supplementary file 1 [file marinedrugs-23-00208-s001.zip › marinedrugs-3595016-supplementary.pdf]

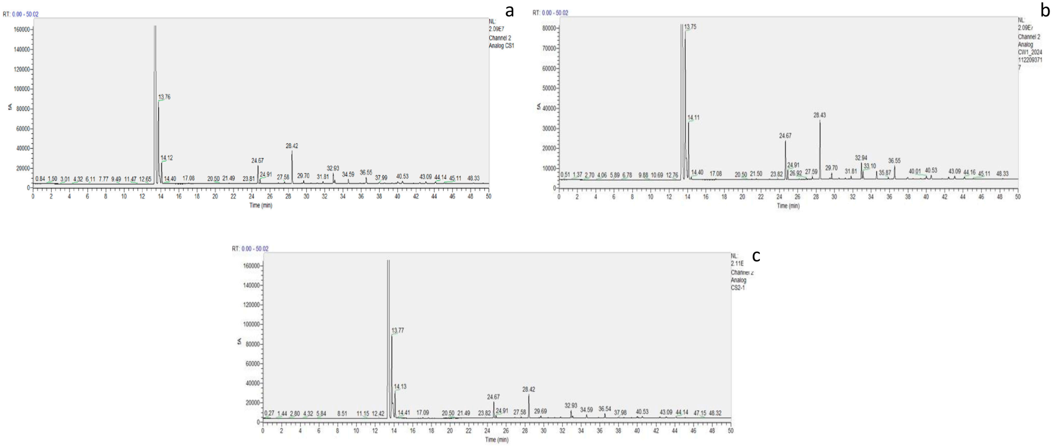

Figure S1. GC-MS chromatograms of the fatty acids (FA) of *C. cylindracea* samples (a: considering CSS1*: Caulerpa* Summer Site 1, b: considering CWS1: *Caulerpa* Winter Site 1 and c: considering CSS2: *Caulerpa* Summer Site 2.
